# Adaptive Mutations in Influenza A/California/07/2009 Enhance Polymerase Activity and Infectious Virion Production

**DOI:** 10.3390/v10050272

**Published:** 2018-05-18

**Authors:** Patrick D. Slaine, Cara MacRae, Mariel Kleer, Emily Lamoureux, Sarah McAlpine, Michelle Warhuus, André M. Comeau, Craig McCormick, Todd Hatchette, Denys A. Khaperskyy

**Affiliations:** 1 Department of Microbiology and Immunology, Dalhousie University, 5850 College Street, Halifax, NS B3H 4R2, Canada; Patrick.Slaine@Dal.Ca (P.D.S.); mariel.kleer@dal.ca (M.K.); craig.mccormick@dal.ca (C.M.); 2 The Hospital for Sick Children, University Health Network, Toronto, ON M5G 2C4, Canada; cara.macrae@mail.utoronto.ca; 3 CGEB-Integrated Microbiome Resource (IMR) and Department of Pharmacology, Dalhousie University, 5850 College Street, Halifax, NS B3H 4R2, Canada; ELamoureux@Dal.Ca (E.L.); Andre.Comeau@Dal.Ca (A.M.C.); 4 Division of Microbiology, Department of Pathology and Laboratory Medicine, Nova Scotia Health Authority (NSHA), Halifax, NS B3H 1V8, Canada; Sarah.McAlpine@Dal.Ca (S.M.); Michelle.Warhuus@iwk.nshealth.ca (M.W.); thatchet@Dal.Ca (T.H.)

**Keywords:** influenza, H1N1, mouse adaptation, deep sequencing, polymerase, PA, PB1, defective viral genomes

## Abstract

Mice are not natural hosts for influenza A viruses (IAVs), but they are useful models for studying antiviral immune responses and pathogenesis. Serial passage of IAV in mice invariably causes the emergence of adaptive mutations and increased virulence. Here, we report the adaptation of IAV reference strain A/California/07/2009(H1N1) (also known as CA/07) in outbred Swiss Webster mice. Serial passage led to increased virulence and lung titers, and dissemination of the virus to brains. We adapted a deep-sequencing protocol to identify and enumerate adaptive mutations across all genome segments. Among mutations that emerged during mouse-adaptation, we focused on amino acid substitutions in polymerase subunits: polymerase basic-1 (PB1) T156A and F740L and polymerase acidic (PA) E349G. These mutations were evaluated singly and in combination in minigenome replicon assays, which revealed that PA E349G increased polymerase activity. By selectively engineering three PB1 and PA mutations into the parental CA/07 strain, we demonstrated that these mutations in polymerase subunits decreased the production of defective viral genome segments with internal deletions and dramatically increased the release of infectious virions from mouse cells. Together, these findings increase our understanding of the contribution of polymerase subunits to successful host adaptation.

## 1. Introduction

Influenza A viruses (IAVs) evolve rapidly and exist as genetically heterogeneous populations known as quasispecies. Water fowl are primary IAV hosts in the wild, but the virus frequently crosses species barriers and adapts to new hosts. There are two primary molecular determinants of rapid IAV evolution and adaptation. First, the IAV genome consists of eight single-stranded RNA segments; co-infection of host cells with two or more genetically-distinct viruses can result in re-assortment of genome segments into hybrid progeny viruses with new properties. This genetic re-assortment is known as antigenic shift [1]. Second, IAV encodes an error-prone RNA-dependent RNA polymerase (RdRp) that misincorporates 2–3 ribonucleotides into each newly-synthesized genome, in a process known as antigenic drift [2]. These processes accelerate viral evolution and allow beneficial mutations to be fixed in the viral genome. Beneficial mutations may increase viral fitness and transmission between hosts in a variety of ways. For example, mutations may help the virus evade host restriction factors or neutralizing antibodies, or confer resistance to antiviral drugs [3,4,5]. Thus, antigenic shift and antigenic drift increase the plasticity of the IAV genome, which enables rapid emergence of viral progeny with new properties [6].

The barriers that limit zoonotic transmission of IAV remain poorly understood, but often involve incompatibilities between viral components and the new host. IAV receptor preferences play an important role in species restriction, with avian hemagglutinin (HA) proteins strongly preferring alpha 2,3 sialic acid receptors, whereas viruses circulating in humans bear adaptive HA mutations that confer efficient binding to alpha 2,6-linked sialic acid receptors present in human airways [7]. Adaptive mutations have also been identified in IAV RdRp proteins. For example, the amino acid residue at position 627 in polymerase basic protein 2 (PB2) has been shown to be an important determinant of host range, with the avian signature glutamic acid at position 627 frequently being substituted for a human signature lysine during mammalian adaptation [8]. The PB2 E627K substitution adapts these viruses for efficient replication in mammalian cells and animal models [9,10]. Reasoning that mammalian cells must lack a necessary host co-factor for avian IAV RdRp activity, Long et al., recently identified an avian protein, ANP32A, as a species-specific co-factor required for efficient avian IAV replication [11].

Inbred mice are relatively inexpensive models for IAV adaptation studies, with readily available reagents and mutant animals [12]. Mice are not natural hosts for IAV; infection with seasonal IAV isolates typically results in an asymptomatic infection with little viral replication. However, most inbred mice are highly susceptible to IAV infection because they lack an interferon-inducible restriction factor known as Mx1 [13,14,15]. Mx1 and its human ortholog MxA inhibit IAV by direct interactions with nucleoprotein (NP) [16,17,18,19,20]. Experimental IAV adaptation to murine hosts requires manual passaging of the virus from infected lungs to naïve hosts, bypassing aerosol transmission [21]. Mouse adaptation is accompanied by increased viral titers in the lung and increased pathogenesis and mortality. In addition to HA mutations that alter sialic acid binding specificity, IAV adaptation in mice has been associated with amino acid substitutions in internal genes. Those include all three polymerase subunits [22,23,24,25,26,27,28], NP [26,27], M1 [29,30] and NS1 [3,31].

Here, we report the adaptation of influenza A/California/07/2009 (CA/07) to an outbred mouse host known as Swiss Webster. Mouse-adapted virus (CA/07-MA) replicated to ~240-times higher titer in the mouse lung than Ca/07 and disseminated to the brain. Comparison of CA/07 and CA/07-MA by deep sequencing revealed several adaptive mutations in polymerase complex proteins polymerase acidic (PA) and polymerase basic-1 (PB1). Interestingly, these substitutions increased polymerase activity in a standard minigenome replication assay and contributed to a 10-fold increase in virion release from mouse cells. Moreover, we observed a ~2.5-fold decrease in the production of defective internally-deleted PB2 genome segments in mouse cells.

## 2. Materials and Methods

### 2.1. Cells and Viruses

Madin-Darby canine kidney (MDCK) cells, human embryonic kidney 293T (HEK293T) cells and mouse L cells were purchased from the American Type Culture Collection (ATCC, Manassas, VA, USA). MDCK, HEK293T, L cells and mouse embryonic fibroblast (MEF, gift from Nancy Kedersha, Brigham and Women’s Hospital, Boston, MA, USA) cells were maintained in Dulbecco’s modified Eagle’s medium (DMEM, HyClone, Mississauga, ON, Canada) supplemented with 10% fetal bovine serum (FBS, Life Technologies, Burlington, ON, Canada) and 20 µM l-glutamine (Life Technologies, Burlington, ON, Canada) at 37 °C in 5% CO_2_ atmosphere. For virus infections, cell monolayers were washed briefly with PBS (Wisent, St-Bruno, QC, Canada) and inoculated with virus diluted in 0.5% bovine serum albumin (BSA, Sigma, St. Louis, MO, USA) DMEM for 1 h at 37 °C with horizontal shaking of the dishes every 10 min. Then, inoculums were removed, monolayers washed with PBS and received fresh 0.5% BSA DMEM supplemented with 20 µM l-glutamine. Influenza A/California/07/2009(H1N1) (CA/07) was provided by the Public Health Agency of Canada (PHAC) National Microbiology Laboratory. CA/07 was plaque-purified in MDCK cells prior to stock preparation in 10-day-old embryonated chicken eggs as described in [32].

### 2.2. Mouse Adaptation of CA/07 Virus

For adaptation experiments, 5–6-week-old outbred female Swiss Webster mice (CFW, Charles River) were used. Animals were treated in accordance with the guidelines of the Canadian Council on Animal Care (CCAC) (Protocol Number 11-006). Prior to infection, mice were anesthetized with isoflurane and then intranasally inoculated with 2 × 10^3^ of 50% tissue culture infectious dose (TCID_50_) units of CA/07 in 50 µL of phosphate buffered saline (PBS). Mice were monitored for weight (with an ethical cut off point of 25% weight loss), ruffled fur, hydration, body temperature and behaviour. Lung tissue was harvested 3 days post infection, homogenised and used for TCID_50_ virus titer determination using the Spearman–Karber method as described in [33]. Fifty microliters of clarified lung homogenate were used for subsequent rounds of infection as described in [21]. After 10 rounds, in addition to lung samples, brain and spleen tissue samples were collected. Mouse-adapted virus (CA/07-MA) was plaque-purified from passage 10 lung homogenates in MDCK cells, and infectious stocks were generated in embryonated chicken eggs as described above for the parental CA/07 virus.

### 2.3. Sequencing and Analysis

Total RNA was isolated from virus-infected MDCK cells using the RNeasy Plus Mini Kit (Qiagen Inc., Toronto, ON, Canada), and the viral genomic RNA was reverse-transcribed as described in [34] using the Maxima H Minus First Strand cDNA Synthesis Kit (Thermo Fisher Scientific, Grand Island, NY, USA) with the Uni12 primer (5′-AGC AAA AGC AGG-3′) [35]. Complimentary DNAs (cDNAs) were amplified for 10 cycles with Phusion High Fidelity DNA Polymerase (NEB) using primers (specific parts underlined) containing Illumina Nextera Transposase adapters: R1-Uni12 (5′-TCG TCG GCA GCG TCA GAT GTG TAT AAG AGA CAG AGC GAA AGC AGG-3′) and R2-Uni13 (5′-GTC TCG TGG GCT CGG AGA TGT GTA TAA GAG ACA GAG TAG AAA CAA GG-3′) using 20-s 48 °C annealing and 7-min 72 °C extension steps (adaptor and barcode oligonucleotide sequences from Illumina, Inc., San Diego, CA, USA). Products were purified using the PCR Purification Kit (Qiagen), and 1 ng was used for Nextera XT (Illumina) library preparation according to the manufacturer’s instructions, with the exception that the kit’s bead-based clean-up and normalization (two steps) were completed instead using the Just-a-Plate 96 PCR Purification and Normalization Kit (CharmBiotech, San Diego, CA, USA) in one step. Complete libraries were pooled and sequenced in a portion of a 300 + 300 bp PE MiSeq run (Illumina 600-cycle v3 kit) by the CGEB-IMR (Centre for Genomics and Evolutionary Biology Integrated Microbiome Resource; (http://cgeb-imr.ca). Raw reads were imported into Geneious R 8.1.8 [36]. Reads were trimmed at default settings, while reads were filtered for a quality (Q) score of 30, selecting reads that have an error probability less than 0.001. Reads were aligned to reference genomes for each individual segment. Once aligned, single nucleotide polymorphisms (SNPs) were identified using Geneious variations/SNPs at 1% abundance [37]. SNP frequencies and locations were imported into R (www.r-project.org) for final analysis. Junctions of internally-deleted viral genomes were called manually in Geneious by identifying incorrectly-aligned reads that spanned the junction. Reads that showed homology to the 3′ and 5′ ends of the viral genome were used to identify the nucleotide positions of the breaks similarly to [38].

### 2.4. Generation of Recombinant Viruses

Eight genomic segments for the parental CA/07 virus were amplified individually from the multisegment cDNA using universal primer sets described in [35] and cloned into the pHW2000 vector [39]. Resulting constructs were named pHW-C71–pHW-C78. Subsequently, T156A and F740L amino acid substitutions were introduced into the PB1 construct to create pHW-C72(T156A,F740L) and E349G in the PA construct to create pHW-C73(E349G) using the Phusion site-directed mutagenesis PCR protocol (NEB). All constructs were verified by Sanger sequencing. Recombinant viruses were rescued from 8 plasmids using HEK293T and MDCK cells as described in [40]. For production of CA/07 virus, the original pHW-C71–pHW-C78 plasmids were used. pHW-C72(T156A,F740L) and pHW-C73(E349G) were substituted for pHW-C72 and pHW-C73 constructs, respectively, to produce CA/07-PA,PB1-MA virus. Both viruses were propagated once in MDCK cells to prepare stocks for subsequent analyses.

### 2.5. Minigenome Assay

Viral RNA polymerase activity was tested in HEK293T cells using the reconstituted minigenome assay with the pPolI-WSN-NA-*firefly*-luciferase reporter construct (gift from Dr. Yoshihiro Kawaoka, University of Wisconsin-Madison, Madison, WI, USA) and in mouse L-cells using the pHL-NS-FF-Luc reporter construct (gift from Dr. Georg Kochs, University of Freiburg, Freiburg, Germany). The assay was performed as described in [41], except the pHW-C71, pHW-C72, pHW-C73 and pHW-C75 plasmids were used for the expression of CA/07 PB2, PB1, PA and NP proteins, respectively, and the pGL4.74(hRluc/TK) plasmid (Promega, Madison, WI, USA) for control Renilla luciferase expression. The dual luciferase assay was performed 24 h post-transfection using the Dual-Glo Luciferase Assay System (Promega). Site-directed mutagenesis was utilised to introduce amino acid substitutions in PB1 and PA expression vectors pHW-C72 and pHW-C73 as described in Section 2.4 above to test their contribution to reconstituted viral polymerase activity.

### 2.6. Immunostaining and Immunoblotting

For immunofluorescence microscopy, cells grown on glass coverslips were fixed and stained as described previously [42] using mouse monoclonal antibody to IAV M1 protein (clone GA2B, AbD SeroTec, Raleigh, NC, USA) and donkey anti-mouse Alexa Fluor-555 conjugated secondary antibody (Molecular Probes, Burlington, ON, Canada). Nuclei were stained with Hoechst dye (Life Technologies, Burlington, ON, Canada). Images were captured using a Zeiss Axioplan II microscope (Zeiss Canada, Toronto, ON, Canada). For Western blotting, whole cell lysates were resolved on denaturing 10% polyacrylamide gels and analyzed using goat polyclonal antibody to influenza A virus (ab20841, Abcam Inc., Toronto, ON, Canada), rabbit anti-PA (GeneTex, Irvine, CA, USA) and β-actin (13E5, HRP-conjugated, NEB).

### 2.7. Real-Time Quantitative PCR

RNA isolation from virus-infected MEF cells and cDNA synthesis were performed as described in Section 2.3 above. Quantitative PCR was performed using GoTaq PCR master mix (Promega) and the following primer pairs: PB2e-Left (5′-GTG CTA ATT GGG CAA GGG GA-3′) and PB2e-Rght (5′-CCA TCC GAA TTC TTT TGG TCG C-3′); PB2i-Left (5′-TGC AAG GCA GCA ATA GGG TT-3′) and PB2i-Rght (5′-AGG TTG CCC GTT AGC ACT TC-3′); NP-Left (5′- GCA ATT CTG CTG CAT TTG AAG AT-3′) and NP-Rght (5′- GCC CAG TAT CTG CTT CTC AGT TC-3′); NS-Left (5′-CTT CGC GCT ACC TTT CTG AC-3′) and NS-Rght (5′-ATT GCT CCC TCC TCA GTG AA-3′). The detailed thermal profile setup and analysis protocols are available upon request.

### 2.8. Plaque Assay

Virion production was determined by the plaque assay in MDCK cells using 1.2% Avicel RC 591 (FMC Corporation, Philadelphia, PA, USA) overlay as described in [43].

### 2.9. Statistical Analysis

Statistical significance was determined using the paired *t* test, with Welch’s correction for standard deviation.

### 2.10. Accession Numbers

Reference sequences used for each segment are as follows; Segment 1-PB2 (NC_026438), Segment 2-PB1 (FJ969531), Segment 3-PA (NC_026437), Segment 4-HA (FJ981613), Segment 5-NP (NC_026436), Segment 6-NA (GQ377078), Segment 7-M (FJ969527) and Segment 8-NS (NC_026432). The protein accession number used for 3D modeling was 4WSB. Images were generated using PyMOL Version 2.0.4 (The PyMOL Molecular Graphics System, Schrödinger, LLC. (http://pymol.sourceforge.net/faq.html)). Mutations were identified in 5 out of 8 segments of the mouse adapted virus. NS, PA, PB2, HA and NP were submitted to GenBank and can be accessed as MG027911, MG027912, MG027913, MG027914 and MG027915, respectively.

## 3. Results

### 3.1. Adaptation of Influenza Strain CA/07/2009 to Swiss Webster Mice

**Figure 1:**
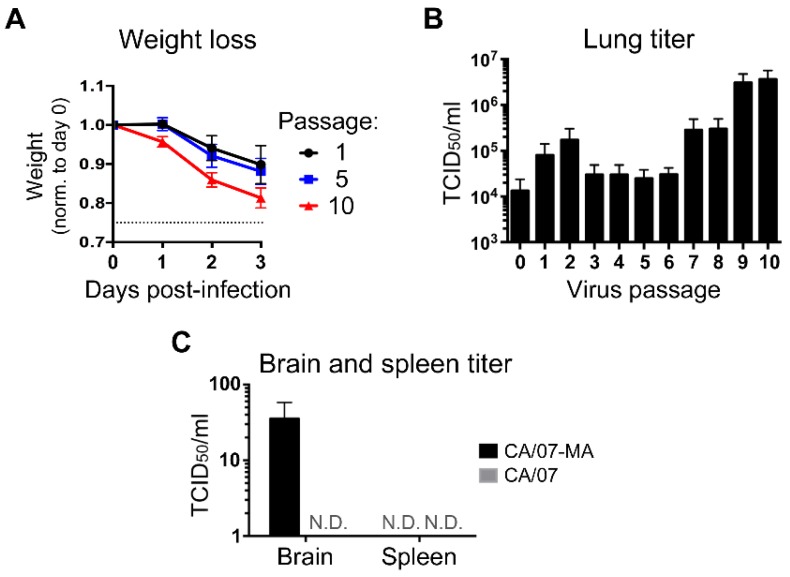
Murine adaptation of CA/07 increases virus replication in lungs and spread to brain. (**A**,**B**) Swiss Webster mice were infected with CA/07, and recovered virus was passaged lung-to-lung nine more times, for a total of ten passages. Morbidity was determined by monitoring weight loss over time ((**A**), passages 1, 5 and 10), and virus titers in the lung were measured by the 50% tissue culture infectious dose (TCID_50_) assay (**B**). (**C**) Dissemination of parental CA/07 and passage 10 (CA/07-MA (mouse adapted)) virus was analyzed by performing TCID_50_ assays on brain and spleen homogenates. N.D. = not detected. In (**A**–**C**), error bars represent the standard deviation (*n* = 4 mice).

Serial passage of seasonal IAV isolates in inbred mice typically causes adaptive mutations that increase virulence [26]. Interestingly, the pandemic H1N1 influenza strain CA/07 has been shown to infect mice and replicate in mouse lungs even before adaptation [44], although the molecular cause of this remains obscure. Here, Swiss Webster mice were serially-infected with 2 × 10^3^ TCID_50_ units of CA/07 to force adaptation to the murine host. At three days post infection, mice were euthanized, and lung tissue was homogenized in PBS to release infectious virions, which were then used to infect the next cohort of mice. After 10 serial passages in naive mice, viruses were harvested for sequencing and phenotypic analysis. As expected, even on the first passage, mice infected with CA/07 displayed clinical symptoms including marked weight loss over the first three days of infection (Figure 1A). TCID_50_ assays conducted on lung homogenates at each passage revealed a greater than two-log increase in viral titer by the ninth passage (Figure 1B). These increased lung titers correlated with accelerated weight loss in mice infected with 10th passage virus (Figure 1A). To measure virus dissemination, samples were taken from the brain and spleen of mice infected with the parental or mouse-adapted virus from passage 10 (hereafter CA/07-MA). We were unable to isolate infectious virus from spleen of mice infected with either virus. However, unlike the parental virus, CA/07-MA could be recovered from brain tissue (Figure 1C). To better understand the immune status of Swiss Webster mice, we amplified and sequenced Mx1 cDNA from passage 10 lung homogenates. Despite their outbred background, these mice had large deletions in both Mx1 alleles (Figure S1). As described previously for inbred mouse strains, these deletions cause frame-shifts in coding sequences that result in premature stop codons generating non-functional Mx1 proteins and unstable Mx1 mRNAs [45]. Thus, while Swiss Webster mice may have fewer defects in innate and adaptive immune responses compared to the commonly-used inbred strains, they are similarly deficient in Mx1 and thus are inherently more susceptible to influenza virus infection.

### 3.2. Identification of CA/07-MA Quasispecies via Deep Sequencing

We used Illumina MiSeq (San Diego, CA, USA) to thoroughly catalog the viral quasispecies that arose during mouse adaptation. In total, parental CA/07 sequencing yielded 1,125,945 reads, compared to 926,042 reads for CA/07-MA. Raw reads were trimmed and aligned to reference sequences (Table 1). Our sequencing methodology enabled maximal coverage of the 5′ and 3′ ends of the viral genome segments, which encompass 3′ untranslated region (UTR) and vRNA packaging sequences. Consistent with previous studies, a fraction of the reads could not be fully aligned to the reference genome, but instead spanned the predicted junctions of internally-deleted viral genomes (DVGs; Table S1). If these DVGs were incorporated into nascent viral particles, they would generate defective-interfering (DI) particles [46,47].

**Figure 2:**
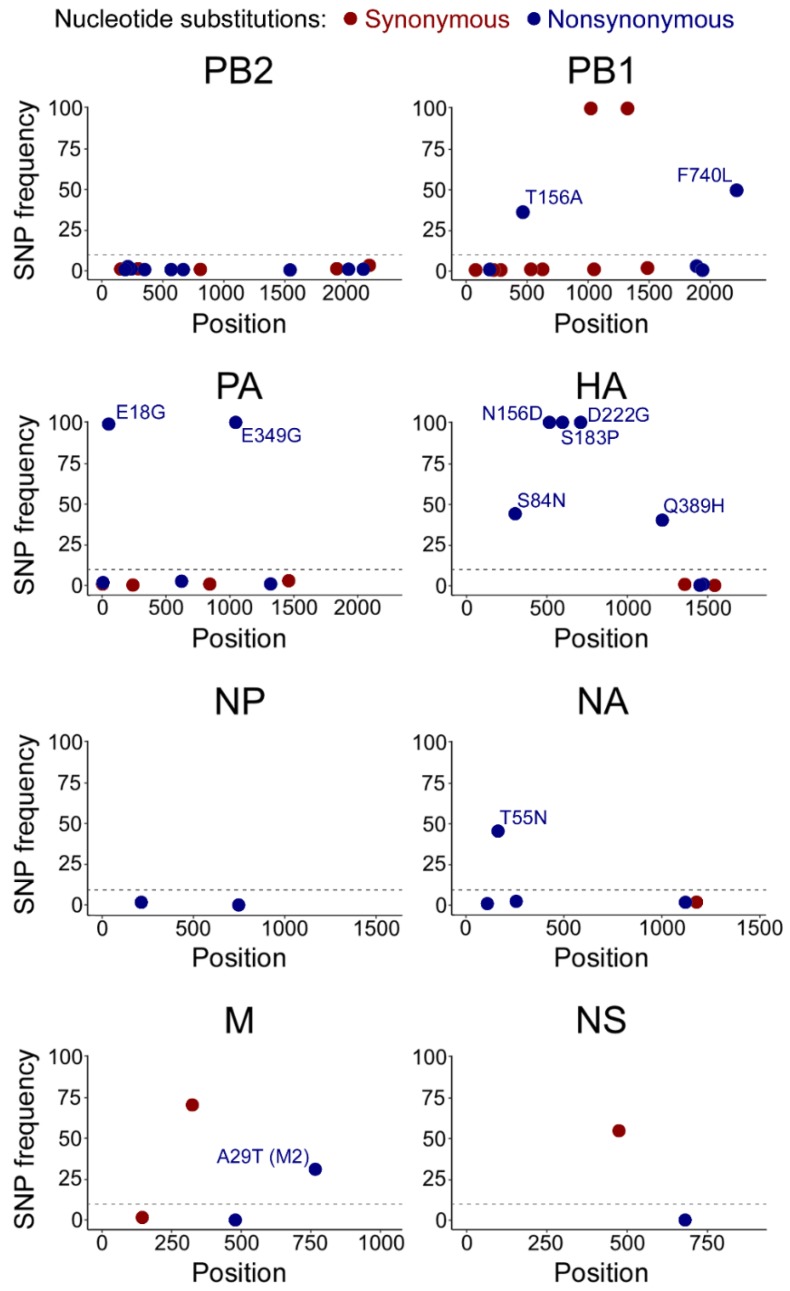
Adaptive substitutions in CA/07-MA identified by deep sequencing. CA/07-MA reads were mapped onto a CA/07 consensus sequence, and substitutions with an increase in frequencies above 1% were plotted. *X*-axis: nucleotide position relative to the adenine of the first AUG start codon in the major open reading frame. *Y*-axis: percent frequency of the substitution. For non-synonymous mutations (blue) with a frequency increase over 10%, the corresponding amino acid change is indicated. SNP: single nucleotide polymorphisms; PB1/2: polymerase basic 1/2; PA: polymerase acidic; HA: hemaglutinin; NP: nucleoprotein; NA: neuraminidase; M: matrix; NS: non-structural.

Using the consensus parental CA/07 sequence as a reference, we identified adaptive mutations and quantified their frequency in the CA/07-MA population. As expected, we observed strong conservation of 5′ and 3′ ends required for vRNA replication and packaging. We also observed four synonymous SNPs in CA/07-MA that did not alter the predicted protein sequence and five nonsynonymous mutations that reached over 50% abundance (Figure 2 and Table S2). Unlike Sanger sequencing, our method allowed us to identify mutations in PB1, HA, NA and M segments, which comprise less than 50% of the population (Table S2). These less abundant missense mutations may contribute to virulence in a swarm of viral quasi-species. Because MiSeq Illumina reads are on average 150 bp in length, it is not possible to know for certain whether these mutations together comprise the dominant genotype.

Among non-synonymous substitutions, HA head domain mutations have previously been shown to increase receptor binding in mouse lungs. We identified three amino acid substitutions in HA that reached >99% frequency in the CA/07-MA strain: N156D, S183P and D222G (Figure 2 and Table S2). Of these substitutions, D222G has been found previously in the two CA/04 mouse adaptation studies [26,27] and is believed to be responsible for increased binding to alpha 2,3-linked sialic acid [48].

**Figure 3:**
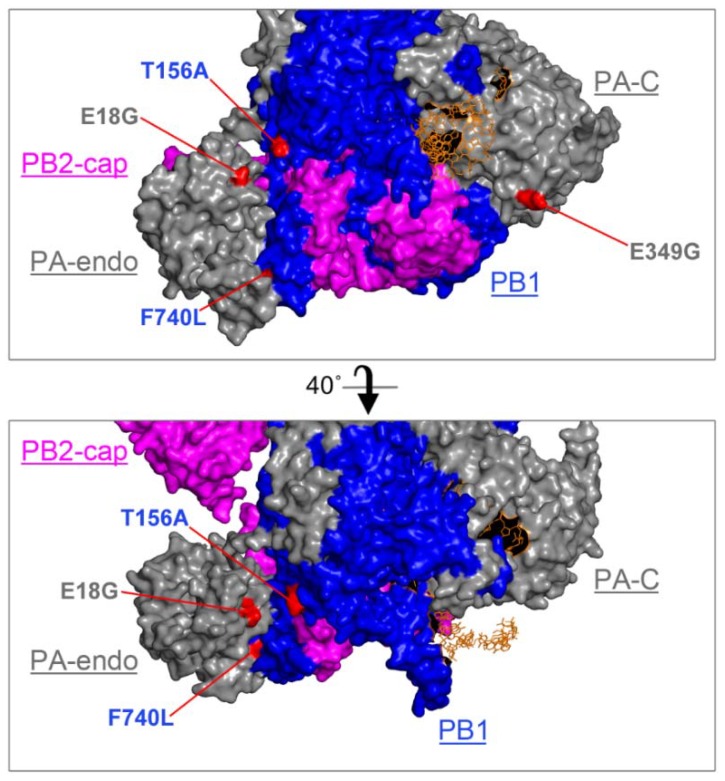
CA/07 mouse adaptation mutations are surface-exposed in the ternary complex. CA/07-MA amino acid substitutions were mapped onto the 3D structure of bat influenza A/little yellow-shouldered bat/Guatemala/060/2010 (H17N10) bound to an RNA primer [49] (protein accession number: 4WSB). PA is grey, PB1 blue, PB2 magenta and the RNA strand orange. Relative positions of the C-terminal cap-binding domain of PB2 (PB2-cap), the N-terminal endonuclease domain of PA (PA-endo) and the C-terminal domain of PA (PA-C) are indicated. Locations of mutations in PB1 and PA identified by deep sequencing are highlighted in red. The image was generated using PyMOL Version 2.0.4. Views were rendered at Ray 2400 with 1000 dpi.

In addition to mutations in HA, we identified four missense mutations in viral polymerase subunits PA and PB1 that exceeded 25% of the population, which we investigated further. In the PA segment, two mutations that resulted in E18G and E349G amino acid substitutions, respectively, reached over 99% in read frequency (Figure 2). In the PB1 segment, two additional substitutions at 36% and 50% frequency resulted in T156A and F740L amino acid substitutions, respectively. We mapped these substitutions onto the only available 3D crystal structure of the IAV polymerase ternary complex from a bat influenza A virus [49]. Despite being separated in the primary sequence, in the 3D structure model, the two PB1 substitutions are in close proximity to each other and the PA interface (Figure 3). The F740L mutation lies within the C-terminal PB2-interacting region of PB1; in the crystal structure, this residue makes direct contact with the PA subunit. Our deep sequencing protocol is limited to generating short ~100–300 bp reads, which prevents us from assigning groups of mutations to a particular genome variant. For this reason, we do not know whether mutations present at <50% frequencies occur in the same molecule, including the F740L and T156A substitutions on PB1. By contrast, the two mutations in PA that reached over 99% abundance must both be present in the majority of genome segments. Interestingly, the PA mutation E18G is located in the amino-terminal endonuclease domain of PA and resides close to PB1 T156A in the 3D structure. The surface-exposed PA E349G substitution is located on the opposite side of the polymerase complex (Figure 3).

### 3.3. Adaptive Substitutions in Polymerase Acidic Protein Enhance Viral RNA Polymerase Activity

**Figure 4:**
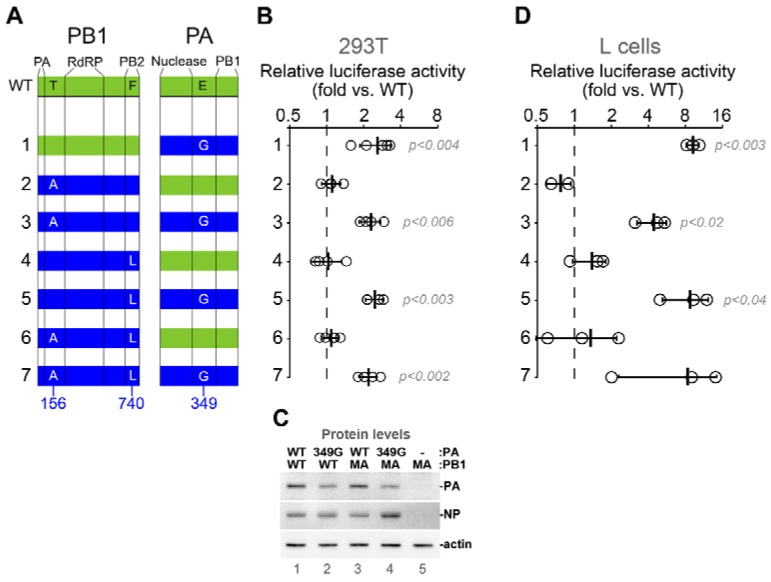
PA E349G substitution enhances viral RNA polymerase activity. (**A**) Schematic representation of PB1 and PA proteins showing approximate boundaries of major domains in the primary sequence (vertical lines) and positions of amino acids mutated in this study. Green and blue shading indicates wild-type and mutant proteins, respectively. (**B**) Relative luciferase activity was measured in the replicon assay using wild-type CA/07-derived PB2 and NP constructs in combination with PB1 and PA constructs that correspond to the numbered combinations depicted in (**A**). Open circles indicate values for each independent replicate normalized to the wild-type replicon values obtained in parallel (dashed line). *p*-values are calculated using the paired Student’s *t* test (*n* ≥ 4). (**C**) Western blot analysis of the whole cell lysates of 293T cells transfected for the select replicon assays shown in (**B**). PB1 and PA variants transfected as indicated. (**D**) The assay described in (**B**) was performed in mouse L cells using a firefly luciferase reporter driven by mouse POL1 promoter. *p*-values are calculated using the paired Student’s *t* test (*n* = 3). RdRP: RNA-dependent RNA polymerase. WT: wild type.

To measure the effects of adaptive mutations on viral polymerase activity in vitro, we tested them individually and in various combinations using a firefly luciferase minireplicon assay in human HEK 293T cells and in mouse L cells (Figure 4). CA/07 PB2, PB1, PA and NP genes of parental CA/07 virus were cloned into expression vectors, and the T156A and F740L substitutions in PB1 and the E349G substitution in PA were subsequently introduced by site-directed mutagenesis. The cloned parental CA/07 PA gene was found to contain glycine at position 18, which was present at only 9.9% frequency in the deep sequenced stock. Since this amino acid was not defined in the reference genome (labelled as X), we decided to leave the G18 in the cloned parental segment unchanged and focus on the three remaining substitutions (Figure 4A). Compared to the reconstituted RNA polymerase complex from parental CA/07 virus, PB1 T156A and PB1 F740L substitutions had no effect on viral RNA polymerase activity when introduced alone or in combination (Figure 4A,B,D). By contrast, the PA E349G substitution increased reporter activity by ~2-fold in 293T cells and by ~10-fold in mouse L cells. Thus, among the three adaptive mutations in PB1 and PA, the E349G substitution had the greatest impact on viral RNA polymerase activity, especially in mouse cells. The effect of E349G substitution in PA was not due to higher expression levels of mutant PA compared to the wild-type PA in this assay (Figure 4C and Figure S2). While other mutations may modulate polymerase activity in vivo, they did not affect polymerase activity in the minireplicon assay.

### 3.4. Adaptive Mutations in Viral Polymerase Subunits PB1 and PA Increase Virus Replication in Mouse Cells

**Figure 5:**
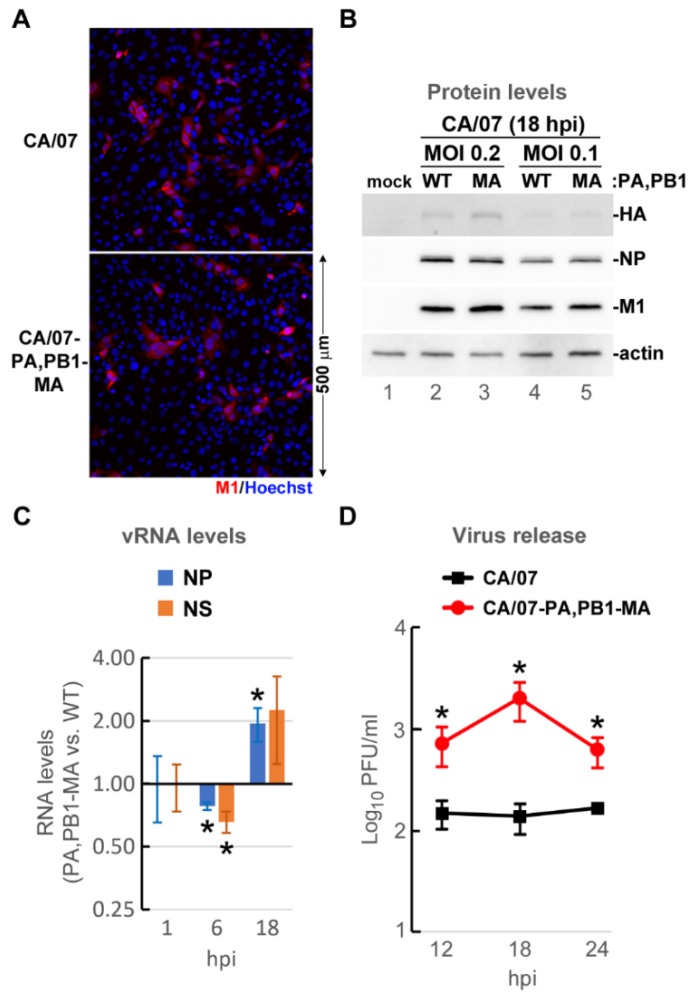
PA and PB1 substitutions increase CA/07 replication in mouse cells. Mouse embryonic fibroblasts were infected with parental CA/07 or recombinant CA/07-PA,PB1-MA viruses at a multiplicity of infection (MOI) of 0.1. (**A**) Cells were fixed at 18 h post infection (hpi) and analysed by immunofluorescence microscopy staining with anti-M1 antibody (red); nuclei were labelled with Hoechst dye (blue). (**B**) Cell lysates harvested at 18 hpi were analysed by Western blotting with antibodies specific for viral M1, NP, HA and cellular actin. (**C**) Total RNA isolated at 1, 6 and 18 hpi was analysed by reverse transcription quantitative PCR (RT-qPCR) to measure levels of influenza A virus (IAV) NP and NS genome segments. Values were normalized to 18S rRNA levels and expressed as the ratio of CA/07-PA,PB1-MA to parental CA/07. (**D**) Virion production was measured at 12, 18 and 24 hpi using the plaque assay in MDCK cells. In (**C**,**D**), error bars represent standard deviations (*n* = 3). * *p*-value < 0.05, paired Student’s *t* test.

To determine whether the CA/07-MA amino acid substitutions that increased minireplicon activity (Figure 4) also affect IAV replication in mouse cells, we created and tested a recombinant CA/07-based virus with three amino acid substitutions: PA E349G and PB1 T156A,F740L (hereafter CA/07-PA,PB1-MA). Mouse embryonic fibroblasts (MEFs) were infected with parental CA/07 or CA/07-PA,PB1-MA recombinant viruses at a multiplicity of infection (MOI) of 0.1 as measured by immunofluorescence staining of infected cells (Figure 5A), and the viral protein accumulation, genome replication and virion production were measured (Figure 5B–D). Although we achieved the same efficiency MEF infection with CA/07 and CA/07-PA,PB1-MA viruses and viral proteins HA, NP and M1 accumulated to comparable levels (Figure 5B), significant differences in viral genome replication were observed between these two viruses. The CA/07-PA,PB1-MA vRNA accumulation was slightly diminished at 6 h post infection (hpi), but was two-fold higher than parental CA/07 by 18 hpi (Figure 5C). At the same time, one-step replication kinetics were markedly accelerated for the CA/07-PA,PB1-MA mutant virus, resulting in 10-fold higher infectious virion release by 18 hpi (Figure 5D).

### 3.5. Adaptive Substitutions in Viral RNA Polymerase Decrease the Production of Defective Viral Genomes

**Figure 6:**
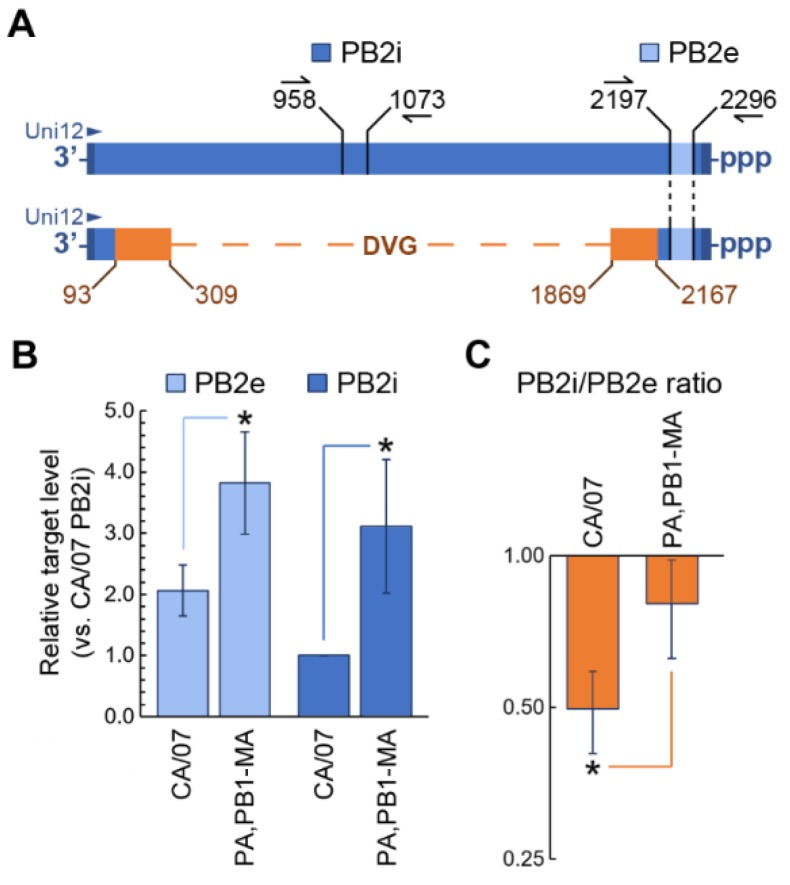
Substitutions in PA and PB1 increase the production of full-length PB2 vRNA. (**A**) Schematic diagram of the full-length PB2 (top) and the PB2-derived defective viral genome (DVG, bottom). In the DVG schematics, regions shared with the full-length segment are colored blue, and the regions containing deletion junction sites are colored orange. Nucleotide positions of the most proximal and most distal junction sites, as determined by deep sequencing, are indicated. Positions of PB2e and PB2i amplicons are depicted, as well as the location of the Uni12 RT primer annealing site (all nucleotide positions are numbered from the full-length vRNA 3′ end). (**B**) Relative levels of total PB2-derived genomic segments (light blue) and the full-length PB2 vRNAs (blue) were measured by RT-qPCR using PB2e and PB2i primer pairs, respectively. (**C**) PB2i to PB2e ratios calculated from (**B**) were plotted. (**B**,**C**) Error bars represent standard deviation (*n* = 3 independent biological replicates). * *p*-value < 0.05, paired Student’s *t* test.

Neither protein nor vRNA accumulation differences could account for 10-fold higher infectious virus production in MEFs infected with CA/07-PA,PB1-MA compared to the wild-type CA/07. Therefore, we sought to determine if the adaptive mutations in viral RNA polymerase genes resulted in reduced accumulation of DVGs. To distinguish between the full-length PB2 vRNA segment and the heterogeneous internally-deleted vRNAs, we designed two qPCR primer pairs. The first pair (PB2e) amplifies the 100-nucleotide region in PB2 vRNA that should be present in both the full-length segment and all the DVGs, as determined by the analysis of junction reads from deep sequencing (described in the Materials and Methods Section and Table S1). The second pair (PB2i) amplifies the 114-nt internal region that is absent in DVGs (Figure 5A). Comparing the levels of these two targets allows us to determine the amounts of full-length PB2 segments and the PB2-derived DVGs in each RNA sample. We measured PB2 vRNA levels at 18 hpi because at this time point, there was much more infectious virus released in CA/07-PA,PB1-MA-infected MEFs compared to parental CA/07 (Figure 5D) and because the PB2 segment was the same between the two viruses. Remarkably, 3 amino acid substitutions in PA and PB1 resulted in roughly 2.5 times more full length PB2 vRNA production (Figure 6B), and the PB2i to PB2e ratio was significantly higher (Figure 6C). This corresponds to a roughly 2.5-times lower frequency of DVG production, which could contribute to considerably higher virion release by CA/07-PA,PB1-MA-infected cells. In theory, packaging of a single DVG segment instead of the full-length one would render the viral particle defective. A similar analysis performed using the parental CA/07 strain and the mouse-adapted CA/07-MA virus stocks showed even larger differences in vRNA replication efficiencies (Figure S3), with roughly 10-times higher levels of full-length PB2 produced in CA/07-MA-infected MEFs at 18 hpi compared to those infected with the parental CA/07 virus (Figure S3C). DVG production was slightly higher in CA/07-MA-infected MEFs, as well (Figure S3D), suggesting that the relationship between RdRp activity and DVG production is not linked solely to the three amino acid changes in PB1 and PA, and other factors could affect the fidelity of vRNA replication.

## 4. Discussion

Following zoonotic transmission, influenza viruses rapidly adapt for optimal replication in a new host [7]. Here, we report the experimental adaptation of the pandemic strain CA/07 to the mouse lung. Most previous adaptation experiments were conducted in inbred mouse strains such as BALB/c and C57BL/6 that have defects in innate immune responses [45]. We selected outbred Swiss Webster mice for influenza adaptation to more faithfully replicate normal murine innate immune responses to infection. Our adaptation protocol was very similar to previously described mouse adaptation studies, wherein virus was serially passaged lung-to-lung [24,26,27,50]. This resulted in increased replication and virulence, as well as dissemination to the brain of infected animals (Figure 1). Due to the substantial increase in virus titers in the lung homogenates from passage 7 onwards, it is possible that the higher infectious dose delivered at passage 10 allowed the virus to reach the brain. Dissemination to the brain was described previously for mouse-adapted A/California/4/09(H1N1) strain (CA/04) [26], which is similar to CA/07. However, neurovirulence is not always linked to mouse adaptation of 2009 pandemic H1N1 viruses; in another report, the increased virulence of CA/04 was restricted to the lungs, and virus was not detected in the brain or other tissues [27]. Both of these previous mouse adaptation experiments were conducted in inbred BALB/c mice using comparable methodologies, so they provide a suitable framework for the discussion of adaptive mutations found in our study.

We utilized Illumina MiSeq deep sequencing to identify adaptive mutations in the CA/07 genome. This methodology allows simultaneous sequencing of all eight IAV genome segments and provides quantitative analysis of mutation frequency; it also provides qualitative and quantitative analysis of DVGs [37,38]. Another advantage of our deep sequencing methodology is that the sample preparation does not require prior plaque purification of the virus, a common step before nucleic acid isolation for Sanger sequencing. Indeed, amplification of virus stocks in eggs or MDCK cells, as well as plaque purification in MDCK cells creates additional bottlenecks for viral quasispecies that may artificially select for variants that grow well in those cell types. For example, our parental CA/07 strain that was amplified in embryonated chicken eggs and plaque-purified in MDCK cells had some of the mutations that were previously attributed to egg or mouse adaptation. Namely, the HA substitutions D222G (at 53.9% frequency, Table S2), S183P (22.8%) and D127E (5.2%) were described in [27], and NP D101G (3.1%) was described in [26,50]. Two of these substitutions reached >99% frequency following serial lung-to-lung passaging in mice and plaque-purification in MDCK cells (D222G and S183P in HA). By contrast, three pre-existing non-synonymous sequence variations were negatively selected in mice and fell below a 1% frequency: HA D127E, NP D101G and NP G102R (Table S2).

Among viral genes that were altered, the HA glycoprotein had the most substitutions (Figure 2). This is not surprising because sialic acid receptors for influenza viruses vary in composition and distribution in different animals. In humans, alpha 2,6-linked sialic acid predominates in the upper respiratory tract. Consequently, HAs of human IAVs, including pandemic H1N1 strains, preferentially bind alpha 2,6-linked sialic acid. By contrast, HAs of avian IAVs bind preferentially alpha 2,3-linked sialic acid found in avian gastrointestinal tracts [51]. The murine respiratory tract also contains alpha 2,3-linked sialic acid [52], and mouse adaptation of human strains usually results in HA mutations that alter sialic acid specificity. A single amino acid substitution D222G was shown to increase binding of HA to alpha 2,3-sialic acid, and we identified this mutation in our CA/07-MA virus. As mentioned above, in the parental virus, this mutation was already present at 53.9% frequency, which increased to 99.5% following mouse lung-to-lung passaging (Table S2). Importantly, the glycine at amino acid position 222 is found in approximately 1% of human H1N1 clinical isolates and is proposed to increase virulence by enhancing binding to alpha 2,3 sialic acid found in the human lower respiratory tract [53]. However, recent studies have challenged this model, suggesting that sialic acid receptor specificity does not necessarily contribute to IAV replication efficiency or virulence in murine and ferret models [54,55]. The HA D222G mutation was also found in the two similar CA/04 mouse adaptation studies [26,27], which distinguishes it from other adaptive mutations in HA that were less reproducible. Of two additional HA mutations that reached >99% frequency in the CA/07-MA strain, S183P substitution was previously identified by Ilyushina et al. [26], while N156D was found only in our study. Another notable HA substitution that appeared at only a 44.5% frequency in CA/07-MA, S84N, was found in human isolates of pandemic H1N1 and was shown to increase in prevalence [56,57,58], yet has not been linked to mouse adaptation so far.

In addition to changes in receptor binding specificity, adaptive mutations in IAV are often located within RNA polymerase segments [7]. In our study, we did not identify adaptive changes in the PB2 gene. At the same time, we observed the emergence of an E349G substitution in PA that reached over 99% read frequency, and two substitutions in PB1: F740L at 49.8% and T156A at 36.3% (Figure 2). We focused our subsequent analyses on these mutations in the RdRp complex to investigate their contribution to mouse adaptation. In the reconstituted viral minireplicon assay, only the PA E349G substitution led to a significant increase in RNA polymerase activity compared to the wild-type (Figure 4). This mutation has been previously identified after sequential passaging of both A/Puerto Rico/8/34 (H1N1) and (A/chicken/Hubei/01/1999) (H9N2) viruses in mice [24,28], and Rolling et al., showed that PA E349G contributed to enhanced polymerase activity and increased titres in the mouse lung following the generation of a recombinant PR8 virus bearing this mutation [24]. This suggests that PA E349G is a marker of mouse adaptation because it dramatically increases minireplicon activity in mouse cells, while the increase in minireplicon activity in human cells is more modest (Figure 4). PA amino acid 349 resides in a domain that was shown to be responsible for viral RdRp association with the host RNA polymerase II through binding to its C-terminal domain [59]. Because mouse and human RNA polymerase II C-terminal domain repeats are identical, it is unclear how position 349 in PA contributes to the enhanced RdRp activity in mouse cells.

Using reverse genetics, we introduced PA and PB1 mutations into the parental CA/07 virus and created the CA/07-PA,PB1-MA virus that differs from the CA/07-MA in that all segments except for PA and PB1, which are identical to the parental strain. Compared to the CA/07 virus, which replicated poorly in MEFs, the CA/07-PA,PB1-MA virus replicated to 10-fold higher titers and produced higher levels of genomic RNA at 18 hpi (Figure 5). Most importantly, infection of MEFs with CA/07-PA,PB1-MA at a low MOI resulted in the generation of much lower levels of DVGs compared to the parental strain (Figure 6). IAV DVGs retain the 5**′** and 3**′** ends of the full-length genome segment, but contain large internal deletions ranging from 180–1000 bp [60]. These deletions occur during replication when the viral polymerase falls off the (+)-sense cRNA template and reattaches a location further downstream [60,61]. Recently, DVGs were detected in specimens collected directly from influenza patients, and their abundance was associated with better clinical outcomes [62].

Previous studies have correlated PA segment mutations with altered DVG production, but the underlying molecular mechanisms remain obscure. The PA A638R mutation was previously linked to an increase in the production of DVGs [63], while the D529N mutation was shown to decrease DVG accumulation [62]. Based on the proximity of amino acids 529 and 638 in existing RdRp structures and the previously identified RNA binding activity of PA, it has been proposed that these residues may function in viral RNA elongation. Available structure models for the trimeric viral RNA polymerase complex suggest that it undergoes large conformational rearrangements in order to switch between cap snatching, transcription and replication functions [64]. Thus, despite its location away from the cluster of residues affecting DVG production, the E349G substitution could affect the efficiency of elongation. Because the E349G substitution arose from several independent mouse adaptation studies, it may be important for RdRp function in mouse cells, potentially via interaction with a host factor, which in turn may influence DVG production.

Recent work has suggested a model of IAV RdRp oligomerization during genome replication and PA residues 293–355 were found to be critical for tetramer formation in silico [65]. Extrapolating from this model, we speculate that the E349G substitution may affect the formation of dimeric and/or tetrameric polymerase subcomplexes. It is not yet known whether RdRp dimerization or tetramerization affects DVG production, or whether host factors may affect oligomerization of the viral RNA polymerase complexes. We speculate that oligomerization could increase polymerase processivity and decrease DVG production. While the mutation of PA residues 351 and 352 to alanines did not affect replication of recombinant viruses [66], a mutation at position 336 has been shown to significantly increase the pathogenicity of CA/04 virus in a mouse model [66]. Future work should address the effects of the C-terminal region of PA in regulating RdRp oligomerization and its effects on polymerase function in different hosts.

Taken together, our findings reveal that adaptive mutations in RdRp subunits increase viral replication efficiency and decrease DVG production. Future studies into the molecular mechanisms that regulate DVG production during IAV infection in different hosts will greatly inform our understanding of IAV pathogenesis and species adaptation.

## Figures and Tables

**Table 1:** Deep sequencing overview for CA/07 and CA/07-MA.

Segment	Length (nt)	CA/07 (Parental)		CA/07-MA	
		Reads	Average Coverage	Reads	Average Coverage
PB2	2341	164,128	9083	238,586	14,561
PB1	2341	246,811	11,777	231,591	16,572
PA	2236	36,512	1615	19,202	1047
HA	1777	144,547	8653	82,826	6377
NP	1565	123,227	7974	81,618	6310
NA	1458	57,397	4654	33,070	2746
M	1027	118,520	11,089	90,245	9089
NS	890	234,803	26,704	148,904	20,738
Total	13,635	1,125,945	10,194	926,042	9680

## Supplementary Materials

The following are available online at http://www.mdpi.com/1999-4915/10/5/272/s1, Table S1: HISAT2 analysis of junction reads, Table S2: Nucleotide substitutions identified by deep sequencing, Figure S1: Outbred Swiss Webster mice have non-functional Mx1, Figure S2: Wild-type and E349G PA are expressed at similar levels in mouse L cells, Figure S3: Efficient replication of the mouse-adapted CA/07-MA virus genome in mouse cells.
