# Supplementary material for: Adaptive Mutations in Influenza A/California/07/2009 Enhance Polymerase Activity and Infectious Virion Production
Source: Viruses. 2018 May 18;10(5):272. doi: 10.3390/v10050272 (PMC5977265; doi:10.3390/v10050272)
Supplement: Supplementary file 1 [file viruses-10-00272-s001.pdf]

Table S1.

Segment	Length (nt)	CA/07 (parental)			CA/07-MA		
		Reads	Junction Reads		Reads	Junction Reads	
			Reads	RPM		Reads	RPM
PB2	2,341	54,846	1,488	27,131	101,081	10,992	108,744
PB1	2,341	78,165	329	4,209	68,778	1,069	15,543
PA	2,236	9,237	36	3,897	4,079	0	0
HA	1,777	41,576	406	9,765	24,717	96	3,884
NP	1,565	42,013	2	48	27,609	0	0
NA	1,458	17,215	0	0	10,275	0	0
M	1,027	40,315	1	25	31,803	0	0
NS	890	86,919	169	1,944	57,800	25	433
total	13,635	370,286	2,431	47,019	326,142	12,182	128,604

HISAT2 analysis of junction reads. Paired reads were trimmed and aligned to reference sequences using HiSat2 to quantify DVG junction-spanning reads. The relative abundance of junction reads was expressed as number of reads per million reads aligned to each segment (RPM). HiSat2 program reference: Bolger, A. M., Lohse, M., & Usadel, B. (2014). Trimmomatic: A flexible trimmer for Illumina Sequence Data. Bioinformatics, btu170.

Table S2.

MA vs Parental										
Segment	Nucleotide #	Reference	Change	Frequency	Log p value	Coverage	ORF	Codon #	Codon change	Substitution
1	162	A	G	0.013	-460	26101	PB2	54	AGA -> AGG	none
1	196	A	G	0.012	-230	15265	PB2	66	AUG -> GUG	M -> V
1	217	C	A	0.032	-597	12107	PB2	73	CAA -> AAA	Q -> K
1	242	C	A	0.014	-128	8440	PB2	81	ACA -> AAA	T -> K
1	244	A	C	0.013	-78	8316	PB2	82	AAC -> CAC	N -> H
1	275	C	G	0.019	-61	2104	PB2	92	UCA -> UGA*	S -> Stop
1	282	G	U	0.013	-21	1696	PB2	94	CUG -> CUU	none
1	309	C	U	0.019	-36	1296	PB2	103	GGC -> GGU	none
1	338	A	G	0.016	-22	1222	PB2	113	AAG -> AGG	K -> R
1	340	G	C	0.013	-18	1269	PB2	114	GUA -> CUA	V -> L
1	353	A	U	0.012	-14	1105	PB2	118	UAU -> UUU	Y -> F
1	575	A	G	0.012	-6	1096	PB2	192	GAG -> GGG	E -> G
1	664	G	A	0.012	-17	1128	PB2	222	GGC -> AGC	G -> S
1	807	A	G	0.013	-6	1235	PB2	269	AGA -> AGG	none
1	1543	C	A	0.010	-17	1758	PB2	515	CCC -> ACC	P -> T
1	1928	C	A	0.015	-40	2020	PB2	643	UCA -> UAA*	S -> Stop
1	2021	C	A	0.019	-119	6880	PB2	674	GCA -> GAA	A -> E
1	2026	A	U	0.011	-68	7278	PB2	676	ACU -> UCU	T -> S
1	2142	C	A	0.014	-326	21255	PB2	714	AGC -> AGA	S -> R
1	2187	G	A	0.038	-2445	40658	PB2	729	GGG -> GGA	none
2	78	A	G	0.010	-323	36526	PB1	26	GGA -> GGG	none
2	200	A	G	0.013	-240	12285	PB1	67	AAC -> AGC	N -> S
2	237	A	C	0.015	-182	10411	PB1	79	CCA -> CCC	none
2	279	U	C	0.014	-101	6695	PB1	93	GCU -> GCC	none
2	466	A	G	0.363	-5947	5829	PB1	156	ACA -> GCA	T -> A
2	1023	C	U	0.996	-23542	6574	PB1	341	CCC -> CCU	none
2	1047	A	U	0.017	-139	6092	PB1	349	GCA -> GCU	none
2	1323	C	A	0.999	-27241	7583	PB1	441	CUC -> CUA	none
2	1891	U	C	0.034	-447	8054	PB1	631	UUU -> CUU	F -> L
2	1934	U	A	0.011	-108	8813	PB1	645	GUA -> GAA	V -> E
2	2218	U	C	0.498	-10861	72743	PB1	740	UUC -> CUC	F -> L
3	7	G	A	0.029	-78	1669	PA	3	GAC -> AAC	D -> N
3	9	C	A	0.023	-58	1632	PA	3	GAC -> GAA	D -> E
3	53	R	G	0.992	-5368	1509	PA	18	GRA -> GGA	X -> G
3	234	A	U	0.011	-8	634	PA	78	AUA -> AUU	none
3	246	A	G	0.011	-6	563	PA	82	AGA -> AGG	none
3	622	A	G	0.033	-26	484	PA	208	ACA -> GCA	T -> A
3	841	A	U	0.015	-11	393	PA	281	AAG -> UAG*	K -> Stop
3	1046	A	G	1.000	-2132	592	PA	349	GAA -> GGA	E -> G
3	1313	U	G	0.014	-9	655	PA	438	AUC -> AGC	I -> S
3	1322	U	G	0.017	-16	647	PA	441	AUG -> AGG	M -> R
4	302	G	A	0.445	-5623	4463	HA	101	AGU -> AAU	S -> N
4	517	A	G	0.997	-12914	3605	HA	173	AAU -> GAU	N -> D
4	598	U	C	0.996	-13820	3971	HA	200	UCU -> CCU	S -> P
4	1218	G	U	0.406	-7006	6212	HA	406	CAG -> CAU	Q -> H
4	1355	U	A	0.015	-114	6240	HA	452	UUG -> UAG	none
4	1454	A	U	0.012	-79	9807	HA	485	UAC -> UUC	Y -> F
4	1460	A	G	0.014	-118	10208	HA	487	AAA -> AGA	K -> R
4	1472	C	A	0.014	-147	11149	HA	491	ACG -> AAG	T -> K
4	1542	A	G	0.010	-100	14220	HA	514	AGA -> AGG	none
5	215	A	C	0.029	-305	7056	NP	72	GAU -> GCU	D -> A
5	748	A	U	0.013	-109	6863	NP	250	AAC -> UAC	N -> Y
6	109	U	C	0.021	-85	2702	NA	37	UCA -> CCA	S -> P
6	164	C	A	0.458	-3778	2799	NA	55	ACU -> AAU	T -> N
6	256	G	A	0.029	-121	2739	NA	86	GCG -> ACG	A -> T
6	1120	A	G	0.027	-122	3085	NA	374	AUU -> GUU	I -> V

6	1173	A	G	0.026	-147	3961	NA	391	CAA -> CAG	none
7	324	G	A	0.705	-23673	10403	M1	108	ACG -> ACA	none
7	479	G	U	0.010	-119	11263	M1	160	CGG -> CUG	R -> L
7	773	G	A	0.314	-16296	18915	M2	29	GCA -> ACA	A -> T
7	832	U	UU	0.022	-117	19986	M2	48	FS**	FS**
8	474	A	G	0.548	-38786	23949	NS1	158	GGA -> GGG	none
8	663	A	G	0.010	-335	37485	NEP	64	AAG -> AGG	K -> R

**MA vs Reference**										
1	162	A	G	0.013	-460	26101	PB2	54	AGA -> AGG	none
1	196	A	G	0.012	-230	15265	PB2	66	AUG -> GUG	M -> V
1	217	C	A	0.032	-597	12107	PB2	73	CAA -> AAA	Q -> K
1	242	C	A	0.014	-128	8440	PB2	81	ACA -> AAA	T -> K
1	244	A	C	0.013	-78	8316	PB2	82	AAC -> CAC	N -> H
1	275	C	G	0.019	-61	2104	PB2	92	UCA -> UGA*	S -> Stop
1	282	G	U	0.013	-21	1696	PB2	94	CUG -> CUU	none
1	309	C	U	0.019	-36	1296	PB2	103	GGC -> GGU	none
1	338	A	G	0.016	-22	1222	PB2	113	AAG -> AGG	K -> R
1	340	G	C	0.013	-18	1269	PB2	114	GUA -> CUA	V -> L
1	353	A	U	0.012	-14	1105	PB2	118	UAU -> UUU	Y -> F
1	575	A	G	0.012	-6	1096	PB2	192	GAG -> GGG	E -> G
1	664	G	A	0.012	-17	1128	PB2	222	GGC -> AGC	G -> S
1	807	A	G	0.013	-6	1235	PB2	269	AGA -> AGG	none
1	1543	C	A	0.010	-17	1758	PB2	515	CCC -> ACC	P -> T
1	1928	C	A	0.015	-40	2020	PB2	643	UCA -> UAA*	S -> Stop
1	2021	C	A	0.019	-119	6880	PB2	674	GCA -> GAA	A -> E
1	2026	A	U	0.011	-68	7278	PB2	676	ACU -> UCU	T -> S
1	2142	C	A	0.014	-326	21255	PB2	714	AGC -> AGA	S -> R
1	2187	G	A	0.038	-2445	40658	PB2	729	GGG -> GGA	none
2	78	A	G	0.010	-323	36526	PB1	26	GGA -> GGG	none
2	200	A	G	0.013	-240	12285	PB1	67	AAC -> AGC	N -> S
2	237	A	C	0.015	-182	10411	PB1	79	CCA -> CCC	none
2	279	U	C	0.014	-101	6695	PB1	93	GCU -> GCC	none
2	466	A	G	0.363	-5947	5829	PB1	156	ACA -> GCA	T -> A
2	1023	C	U	0.996	-23542	6574	PB1	341	CCC -> CCU	none
2	1047	A	U	0.017	-139	6092	PB1	349	GCA -> GCU	none
2	1323	C	A	0.999	-27241	7583	PB1	441	CUC -> CUA	none
2	1891	U	C	0.034	-447	8054	PB1	631	UUU -> CUU	F -> L
2	1934	U	A	0.011	-108	8813	PB1	645	GUA -> GAA	V -> E
2	2218	U	C	0.498	-10861	72743	PB1	740	UUC -> CUC	F -> L
3	7	G	A	0.029	-78	1669	PA	3	GAC -> AAC	D -> N
3	9	C	A	0.023	-58	1632	PA	3	GAC -> GAA	D -> E
3	53	R	G	0.992	-5368	1509	PA	18	GRA -> GGA	X -> G
3	234	A	U	0.011	-8	634	PA	78	AUA -> AUU	none
3	246	A	G	0.011	-6	563	PA	82	AGA -> AGG	none
3	622	A	G	0.033	-26	484	PA	208	ACA -> GCA	T -> A
3	841	A	U	0.015	-11	393	PA	281	AAG -> UAG*	K -> Stop
3	1046	A	G	1.000	-2132	592	PA	349	GAA -> GGA	E -> G
3	1313	U	G	0.014	-9	655	PA	438	AUC -> AGC	I -> S
3	1322	U	G	0.017	-16	647	PA	441	AUG -> AGG	M -> R
4	302	G	A	0.445	-5623	4463	HA	101	AGU -> AAU	S -> N
4	517	A	G	0.997	-12914	3605	HA	173	AAU -> GAU	N -> D
4	598	U	C	0.996	-13840	3971	HA	200	UCU -> CCU	S -> P
4	716	A	G	0.995	-14169	4083	HA	239	GAU -> GGU	D -> G
4	1218	G	U	0.406	-7006	6212	HA	406	CAG -> CAU	Q -> H
4	1355	U	A	0.015	-114	6240	HA	452	UUG -> UAG	none
4	1454	A	U	0.012	-79	9807	HA	485	UAC -> UUC	Y -> F
4	1460	A	G	0.014	-118	10208	HA	487	AAA -> AGA	K -> R
4	1472	C	A	0.014	-147	11149	HA	491	ACG -> AAG	T -> K
4	1542	A	G	0.010	-100	14220	HA	514	AGA -> AGG	none

5	159	U	G	0.996	-25567	7357	NP	53	GAU -> GAG	D -> E
5	215	A	C	0.029	-305	7056	NP	72	GAU -> GCU	D -> A
5	341	R	A	0.998	-25499	7109	NP	114	GRA -> GAA	X -> E
5	365	U	A	0.999	-25866	7195	NP	122	CUA -> CAA	L -> Q
5	748	A	U	0.013	-109	6863	NP	250	AAC -> UAC	N -> Y
6	109	U	C	0.021	-85	2702	NA	37	UCA -> CCA	S -> P
6	164	C	A	0.458	-3778	2799	NA	55	ACU -> AAU	T -> N
6	256	G	A	0.029	-121	2739	NA	86	GCG -> ACG	A -> T
6	1052	A	U	0.998	-10876	3115	NA	351	UAC -> UUC	Y -> F
6	1059	U	C	0.996	-11294	3157	NA	353	UAU -> UAC	none
6	1120	A	G	0.027	-122	3085	NA	374	AUU -> GUU	I -> V
6	1173	A	G	0.026	-147	3961	NA	391	CAA -> CAG	none
7	324	G	A	0.705	-23673	10403	M1	108	ACG -> ACA	none
7	479	G	U	0.010	-119	11263	M1	160	CGG -> CUG	R -> L
7	773	G	A	0.314	-16296	18915	M2	29	GCA -> ACA	A -> T
7	832	U	UU	0.022	-117	19986	M2	48	FS**	FS**
8	474	A	G	0.548	-38786	23949	NS1	158	GGA -> GGG	none
8	663	A	G	0.010	-335	37485	NEP	64	AAG -> AGG	K -> R

**Parental vs Reference**										
1	207	G	C	0.018	-237	8165	PB2	69	GAG -> GAC	E -> D
1	265	G	C	0.011	-49	3218	PB2	89	GUG -> CUG	V -> L
1	462	C	U	0.022	-55	1690	PB2	154	CUC -> CUU	none
1	696	G	A	0.015	-37	1832	PB2	232	UUG -> UUA	none
1	1073	A	C	0.015	-14	1863	PB2	358	GAA -> GCA	E -> A
1	1311	U	C	0.013	-40	2356	PB2	437	CAU -> CAC	none
1	1445	A	G	0.014	-46	2385	PB2	482	AAA -> AGA	K -> R
1	1711	U	C	0.163	-1198	3036	PB2	571	UUA -> CUA	none
1	2052	U	C	0.017	-272	15001	PB2	684	UCU -> UCC	none
2	760	U	C	0.016	-218	9780	PB1	254	UUU -> CUU	F -> L
2	1023	C	U	0.297	-8748	10899	PB1	341	CCC -> CCU	none
2	1464	G	A	0.067	-1444	10629	PB1	488	GGG -> GGA	none
3	53	R	A	0.898	-8660	2801	PA	18	GRA -> GAA	X -> E
3	53	R	G	0.099	-607	2801	PA	18	GRA -> GGA	X -> G
3	183	A	U	0.011	-20	1501	PA	61	AUA -> AUU	none
3	228	U	C	0.014	-20	1051	PA	76	UUU -> UUC	none
3	1848	G	A	0.011	-26	1981	PA	616	UCG -> UCA	none
4	165	C	A	0.019	-204	7500	HA	55	AAC -> AAA	N -> K
4	166	G	A	0.012	-113	7493	HA	56	GGG -> AGG	G -> R
4	171	A	U	0.018	-192	7337	HA	57	AAA -> AAU	K -> N
4	172	C	A	0.013	-116	7424	HA	58	CUA -> AUA	L -> I
4	424	A	G	0.473	-8102	5776	HA	142	AAU -> GAU	N -> D
4	432	C	A	0.052	-567	5881	HA	144	GAC -> GAA	D -> E
4	437	A	G	0.068	-788	5719	HA	146	AAC -> AGC	N -> S
4	511	A	G	0.039	-395	5370	HA	171	AAA -> GAA	K -> E
4	598	U	C	0.228	-3613	6127	HA	200	UCU -> CCU	S -> P
4	622	C	A	0.101	-1362	6144	HA	208	CUC -> AUC	L -> I
4	716	A	G	0.539	-10487	6609	HA	239	GAU -> GGU	D -> G
4	719	A	G	0.018	-173	6657	HA	240	CAA -> CGA	Q -> R
4	1433	G	A	0.022	-335	10568	HA	478	GGA -> GAA	G -> E
5	159	U	G	0.676	-17278	7995	NP	53	GAU -> GAG	D -> E
5	302	A	G	0.031	-479	9687	NP	101	GAC -> GGC	D -> G
5	304	G	A	0.265	-6784	9637	NP	102	GGA -> AGA	G -> R
5	324	C	U	0.270	-7248	10439	NP	108	CUC -> CUU	none
5	341	R	A	0.993	-36918	10377	NP	114	GRA -> GAA	X -> E
5	365	U	A	0.998	-38140	10620	NP	122	CUA -> CAA	L -> Q
6	17	A	G	0.011	-37	2947	NA	6	AAG -> AGG	K -> R
6	258	G	A	0.019	-113	3863	NA	86	GCG -> GCA	none
6	1052	A	U	0.996	-19144	5347	NA	351	UAC -> UUC	Y -> F
6	1059	U	C	0.996	-19523	5455	NA	353	UAU -> UAC	none

7	177	U	C	0.012	-202	14062	M1	59	AUU -> AUC	none
8	42	C	U	0.019	-806	31645	NS1	14	UUC -> UUU	none
8	214	G	A	0.026	-1476	37724	NS1	72	GAA -> AAA	E -> K
8	355	U	C	0.018	-991	38197	NS1	119	UUG -> CUG	none
8	817	U	C	0.010	-459	38088	NEP	115	GCU -> GCC	none

Nucleotide substitutions identified by deep sequencing. Sequencing reads were aligned to reference genomes for each segment as described in materials and methods section. Differences between the mouse-adapted CA/07-MA sequence and parental CA/07 sequence (MA vs Parental), the mouse-adapted CA/07-MA and reference sequence (MA vs Reference), and the parental CA/07 and reference sequence (Parental vs Reference) found at frequencies above 0.01 are presented in the table.

A

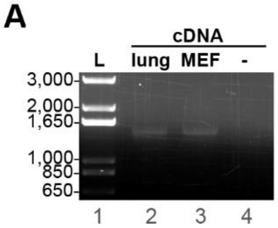

B

```
Mx1_Reference                                                 ATGGATTCTGTGAATAATCTGTGCAGG 240
Mx1_copy1                                                     NNNNNNNNNNNNNNNNNNNNNNNNNNN 27
Mx1_copy2                                                     NNNNNNNNNNNNNNNNNNNNNNNNNNN 27

Mx1_Reference CACTATGAGGAGAAGGTGCGGCCCTGTATTGACCTCATCGACACCCTGAGGGCTCTGGGT 300
Mx1_copy1     NNNNNNNNNNNNNNNNNGCGGCCCTGTATTGACCTCATCGACACCCTGAGGGCTCTGGGT 87
Mx1_copy2     NNNNNNNNNNNNNNNNNGCGGCCCTGTATTGACCTCATCGACACCCTGAGGGCTCTGGGT 87
                               *******************************************

Mx1_Reference GTGGAGCAGGACCTGGCCCTGCCTGCCATCGCTGTCATTGGGGACCAGAGTTCAGGGAAG 360
Mx1_copy1     GTGGAGCAGGACCTGGCCCTGCCTGCCATCGCTGTCATTGGGGACCAGAGTTCAGGGAAG 147
Mx1_copy2     GTGGAGCAGGACCTGGCCCTGCCTGCCATCGCTGTCATTGGGGACCAGAGTTCAGGGAAG 147
              ************************************************************

Mx1_Reference AGCTCTGTTCTGGAAGCACTGTCTGGAGTGGCCCTCCCCAGAGGCAGTGGTATTGTCACC 420
Mx1_copy1     AGCTCTGTTCTGGAAGCACTGTCTGGAGTGGCCCTCCCCAGAGGCAGTGGTATTGTCACC 207
Mx1_copy2     AGCTCTGTTCTGGAAGCACTGTCTGGAGTGGCCCTCCCCAGAGGCAGTGGTATTGTCACC 207
              ************************************************************

Mx1_Reference AGATGCCCTCTGGTGCTGAAATTGAGGAAGCTGAAAGAAGGAGAGGAGTGGAGAGGCAAA 480
Mx1_copy1     AGATGCCCTCTGGTGCTGAAATTGAGGAAGCTGAAAGAAGGAGAGGAGTGGAGAGGCAAA 267
Mx1_copy2     AGATGCCCTCTGGTGCTGAAATTGAGGAAGCTGAAAGAAGGAGAGGAGTGGAGAGGCAAA 267
              ************************************************************

Mx1_Reference GTCTCCTATGATGACATCGAAGTGGAGCTCTCTGATCCTTCAGAGGTGGAAGAGGCCATT 540
Mx1_copy1     GTCTCCTATGATGACATAGAAGTGGAGCTCTCTGATCCTTCAGAGGTGGAAGAGGCCATC 327
Mx1_copy2     GTCTCCTATGATGACATAGAAGTGGAGCTCTCTGATCCTTCAGAGGTGGAAGAGGCCATC 327
              ***************** ****************************************** 

Mx1_Reference AACAAGGGTCAGAACTTCATTGCTGGGGTAGGCCTGGGGATCAGTGATAAGCTCATTAGC 600
Mx1_copy1     AACAAGGGTCAGAACTTCATTGCTGGGGTAGGCCTGGGGATCAGTGATAAGCTCATTAGC 387
Mx1_copy2     AACAAGGGTCAGAACTTCATTGCTGGGGTAGGCCTGGGGATCAGTGATAAGCTCATTAGC 387
              ************************************************************

Mx1_Reference CTGGATGTCAGCTCCCCAAATGTCCCAGACCTGACTCTCATTGACCTGCCTGGAATTACC 660
Mx1_copy1     CTGGATGTCAGCTCCCCAAATGTCCCAGACCTGACTCTCATTGACCTGCCTGGAATTACC 447
Mx1_copy2     CTGGATGTCAGCTCCCCAAATGTCCCAGACCTGACTCTCATTGACCTGCCTGGAATTACC 447
              ************************************************************

Mx1_Reference AGGGTGGCTGTAGGCAACCAGCCTGCAGACATAGGACGCCAGATCAAGAGACTTATCAAG 720
Mx1_copy1     AGGGTGGCTGTAGGCAACCAGCCTGCAGACATAGGACGCCAGATCAAGAGACTTATCAAG 507
Mx1_copy2     AGGGTGGCTGTAGGCAACCAGCCTGCAGACATAGGACGCCAGATCAAGAGACTTATCAAG 507
              ************************************************************

Mx1_Reference ACATACATCCAAAAACAAGAGACCATCAACCTGGTGGTAGTCCCAGCAATGTGGACATT 780
Mx1_copy1     ACATACATCCAAAAACAAGAGACCATCAACCTGGTGGTAGTCCCAGTAATGTGGACATT 567
Mx1_copy2     ACATACATCCAAAAACAAGAGACCATCAACCTGGTGGTAGTCCCAGTAATGTGGACATT 567
              ********************************************** *************

Mx1_Reference GCTACCACAGAGGCTCTGAGCATGGCTCAGGAGGTGGACCCTGAAGGGGATAGGACCATA 840
Mx1_copy1     GCTACCACAGAGGCTCTGAGCATGGCTCAGGAGGTGGACCCTGAAGGGGATAGGACCATA 627
Mx1_copy2     GCTACCACAGAGGCTCTGAGCATGGCTCAGGAGGTGGACCCTGAAGGGGATAGGACCATA 627
              ************************************************************

Mx1_Reference GGGGTCTTGACCAAGCCTGATCTGGTGGACAGAGGTGCTGAAGGCAAGGTCTTGGATGTG 900
Mx1_copy1     GGGGTCTTGACCAAGCCTGATCTGGTGGACAGAGGTGTTGAAGGCAAGGTCTTGGATGTG 687
Mx1_copy2     GGGGTCTTGACCAAGCCTGATCTGGTGGACAGAGGTGTTGAAGGCAAGGTCTTGGATGTG 687
              ************************************* **********************

Mx1_Reference ATGCGGAACCTGGTGTATCCTCTGAAGAAGGGCTACATGATAGTCAAGTGCAGAGGTCAG 960
Mx1_copy1     ATGCGGAACCTGGTGTATCCTCTGAAGAAGGGCTACATGATAGTCAAGTGCAGAGGTCAG 747
Mx1_copy2     ATGCGGAACCTGGTGTATCCTCTGAAGAAGGGCTACATGATAGTCAAGTGCAGAGGTCAG 747
              ************************************************************

Mx1_Reference CAGGACATCCAAGAGCAGCTGAGCCTGACTGAGGCTTTTCAGAAAGAGCAAGTCTTCTTC 1020
Mx1_copy1     CAGGACATTCAAGAGCAGCTGAGCCTGACTGAGGCTTTTCAGAAAGAGCAAGTCTTCTTC 807
Mx1_copy2     CAGGACATTCAAGAGCAGCTGAGCCTGACTGAGGCTTTTCAGAAAGAGCAAGTCTTCTTC 807
              ******** ***************************************************

Mx1_Reference AAGGATCACTCATACTTCAGCATTCTTCTGGAAGATGGGAAGGCCACAGTGCCCTGCCTG 1080
Mx1_copy1     AAGGATCACTCATACTTCAGCATTCTTCTGGAAGATGGGAAGGCCACAGTGCCCTGCCTG 867
Mx1_copy2     AAGGATCACTCATACTTCAG---------------------------------------- 827
              ********************

Mx1_Reference GCAGAGAGACTGACTGAGGAGCTCACCTCCCACATCTGTAAATCACTGCCACTATTGGAA 1140
Mx1_copy1     GCAGAGAGACTGACTGAGGAGCTCACCTCCCACATCTGT--------------------- 906
Mx1_copy2     ------------------------------------------------------------ 827

Mx1_Reference GATCAAATAAATAGCAGTCATCAGAGTGCAAGCGAGGAGCTGCAGAAGTACGGTGCAGAC 1200
Mx1_copy1     ------------------------------------------------------------ 906
Mx1_copy2     ------------------------------------------------------------ 827

Mx1_Reference ATACCAGAAGATGACAGAACGAGGATGTCCTTTCTGGTGAATAAAATCAGTGCCTTCAAT 1260
Mx1_copy1     ------------------------------------------------------------ 906
Mx1_copy2     ------------------------------------------------------------ 827

Mx1_Reference AGGAATATCATGAATTTGATACAAGCACAGGAAACCGTATCAGAGGGAGACAGCCGGTTG 1320
Mx1_copy1     ------------------------------------------------------------ 906
Mx1_copy2     ------------------------------------------------------------ 827

Mx1_Reference TTTACCAAACTGCGAAATGAGTTTCTTGCTTGGGATGATCATATTGAGGAATATTTTAAA 1380
Mx1_copy1     ------------------------------------------------------------ 906
Mx1_copy2     ------------------------------------------------------------ 827

Mx1_Reference AAAGATTCTCCTGAGGTACAAAGCAAGATGAAAGAATTTGAAAATCAGTATCGTGGCCGG 1440
Mx1_copy1     ------------------------------------------------------------ 906
Mx1_copy2     ------------------------------------------------------------ 827

Mx1_Reference GAGCTGCCAGGGTTTGTGGACTACAAGGCATTTGAGAGCATCATCAAAAAGCGAGTCAAA 1500
Mx1_copy1     ------------------------------------------------------------ 906
Mx1_copy2     ------------------------------------------------------------ 827

Mx1_Reference GCCCTGGAAGAGTCTGCTGTGAACATGCTGCGCAGGGTCACTAAGATGGTCCAAACTGCC 1560
Mx1_copy1     -------------------------------------------AGATGGTCCAAACTGCC 923
Mx1_copy2     -------------------------------------------AGATGGTCCAAACTGCC 844
                                                         *****************

Mx1_Reference TTCGTAAAGATTTTATCAAATGATTTTGGTGATTTTTTAAACCTCTGCTGTACTGCTAAG 1620
Mx1_copy1     TTCGTAAAGATTTTATCAAATGATTTTGGTGATTTTTTAAACCTCTGCTGTACTGCTAAG 983
Mx1_copy2     TTCGTAAAGATTTTATCAAATGATTTTGGTGATTTTTTAAACCTCTGCTGTACTGCTAAG 904
              ************************************************************

Mx1_Reference TCCAAAATTAAAGAAATAAGATTAAACCAAGAAAAGGAAGCTGAAAACCTGATCCGACTT 1680
Mx1_copy1     TCCAAAATTAAAGAAATAAGATTAAACCAAGAAAAGGAAGCTGAAAACCTGATCCGACTT 1043
Mx1_copy2     TCCAAAATTAAAGAAATAAGATTAAACCAAGAAAAGGAAGCTGAAAACCTGATCCGACTT 964
              ************************************************************

Mx1_Reference CACTTCCAGATGGAACAGATTGTCTACTGCCAGGACCAGGTTTACAAGGAAACCTTGAAG 1740
Mx1_copy1     CACTTCCAGATGGAACAGATTGTCTACTGCCAGGACCAGGTTTACAAGGAAACCTTGAAG 1103
Mx1_copy2     CACTTCCAGATGGAACAGATTGTCTACTGCCAGGACCAGGTTTACAAGGAAACCTTGAAG 1024
              ************************************************************

Mx1_Reference ACGATCAGAGAGAAGGAAGCTGAGAAAGAGAAGACCAAGGCATTAATAAACCCTGCTACC 1800
Mx1_copy1     ACGATCAGAGAGAAGGAAGCTGAGAAAGAGAAGACCAAGGCATTAATAAACCCTGCTACC 1163
Mx1_copy2     ACGATCAGAGAGAAGGAAGCTGAGAAAGAGAAGACCAAGGCATTAATAAACCCTGCTACC 1084
              ************************************************************

Mx1_Reference TTTCAAAATAACTCTCAGTTTCCTCAAAAGGGGTTGACTACCACTGAGATGACCCAGCAC 1860
Mx1_copy1     TTTCAAAATAACTCTCAGTTTCCTCAAAAGGGGTTGACTACCACTGAGATGACCCAGCAC 1223
Mx1_copy2     TTTCAAAATAACTCTCAGTTTCCTCAAAAGGGGTTGACTACCACTGAGATGACCCAGCAC 1144
              ************************************************************

Mx1_Reference CTGAAAGCCTACTACCAGGAGTGCAGACGGAATATTGGGAGACAGATCCCTCTGATTATC 1920
Mx1_copy1     CTGAAAGCCTACTACCAGGAGTGCAGACGGAATATTGGGAGACAGATCCCTCTGATTATC 1283
Mx1_copy2     CTGAAAGCCTACTACCAGGAGTGCAGACGGAATATTGGGAGACAGATCCCTCTGATTATC 1204
              ************************************************************

Mx1_Reference CAGTACTTCATCCTGAAAACATTTGGGGAGGAAATAGAGAAAATGATGCTTCAGCTTTTA 1980
Mx1_copy1     CAGTACTTCATCCTGAAAACATTTGGGGAGGAAATAGAGAAAACGATGCTTCAGCTTTTA 1343
Mx1_copy2     CAGTACTTCATCCTGAAAACATTTGGGGAGGAAATAGAGAAAACGATGCTTCAGCTTTTA 1264
              ******************************************* ****************

Mx1_Reference CAGGACACCAGTAAGTGCAGCTGGTTCCTGGAGGAGCAGAGTGACACCAGAGAGAAGAAG 2040
Mx1_copy1     CAGGACACCAGTAAGTGCAGCTGGTTCCTGGAGGAGCAGAGTGACACCAGAGAGAAGAAG 1403
Mx1_copy2     CAGGACACCAGTAAGTGCAGCTGGTTCCTGGAGGAGCAGAGTGACACCAGAGAGAAGAAG 1324
              ************************************************************

Mx1_Reference AAGTTCCTGAAAAGGCGGCTTTTAAGGCTGGATGAGGCTCGGCAGAAGCTTGCCAAATTC 2100
Mx1_copy1     AAGTTCCTGAAGAGGCGGCTTTTAAGGCTGGATGAGGCTCGGCAGAAGCTTGCCAAANNN 1463
Mx1_copy2     AAGTTCCTGAAGAGGCGGCTTTTAAGGCTGGATGAGGCTCGGCAGAAGCTTGCCAAANNN 1384
              *********** *********************************************

Mx1_Reference TCCGATTAA 2109
Mx1_copy1     NNNNNNNNN 1472
Mx1_copy2     NNNNNNNNN 1393
```

Figure S1. Outbred Swiss Webster mice have non-functional Mx1. Total RNA was extracted from a Swiss Webster mouse lung homogenate collected at 3 days post-infection with passage 10 CA/07-MA virus (lung) and from mouse embryonic fibroblasts originating from C57BL/6 strain (MEF). (A) Following reverse transcription using Maxima H Minus reverse transcriptase (Thermo) and oligo(dT) priming the coding sequences of Mx1 cDNA were amplified and the products were resolved on 1% agarose gel stained with ethidium bromide (expected product size for the full-length Mx1 coding region is 1896 bp). PCR product from lane 2 (lung) was gel-extracted and sequenced. Sequencing revealed that the gel-extracted band contained two Mx1 amplicons: 1472 bp and 1393 bp

that were not resolved by electrophoresis. Both sequences were aligned to the reference Mx1 sequence (NM_010846) using Clustal Omega (https://www.ebi.ac.uk/Tools/msa/clustalo/) and the portions of the alignment corresponding to the full-length Mx1 coding sequence are presented in (B). Sequences that correspond to primers used for amplification are underlined. The start and stop codons for the full-length Mx1 are highlighted green. Premature stop codons that resulted from deletions in both copies of Mx1 amplified from Swiss Webster mouse lung cDNA are highlighted red.

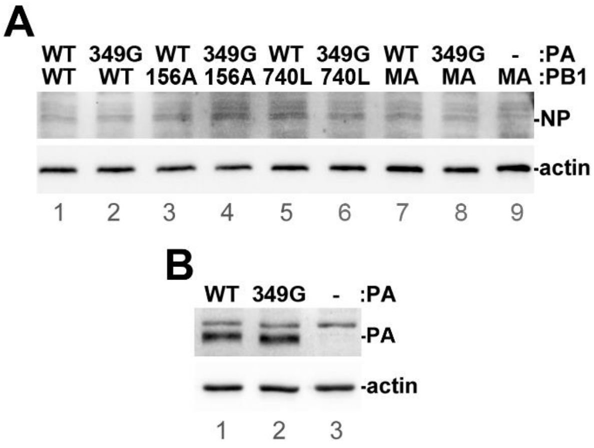

Figure S2. Wild-type and E349G PA are expressed at similar levels in mouse L cells. (A) Western blot analysis of the whole cell lysates of L cells transfected with 5 plasmids encoding the reconstituted minireplicon and the control pGL4.74(hRluc/TK) plasmid. PB1 and PA variants compared in the minireplicon assay are indicated. Due to low levels of protein expression from pHW vectors in a 6-vector mix, very low signal for NP could be detected in lanes 1-8 and no signal for PA could be detected (not shown). (B) Western blot analysis of the whole cell lysates of L cells transfected with either wild-type (WT) or E349G mutant (349G) pHW-PA expression vectors alone, or the empty vector control (-), showing comparable expression levels of PA proteins.

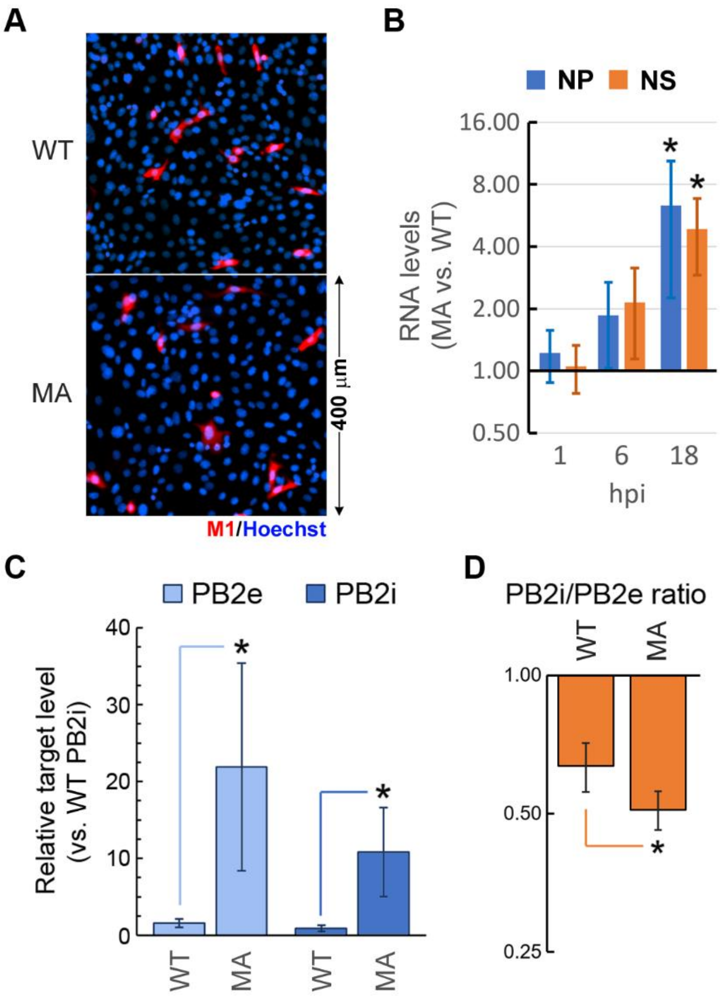

Figure S3. Efficient replication of the mouse-adapted CA/07-MA virus genome in mouse cells. Mouse embryonic fibroblasts were infected with parental CA/07 or the mouse-adapted CA/07-MA viruses at an MOI of 0.03. A) Cells were fixed at 18 hpi and analysed by immunofluorescence microscopy staining with anti-M1 antibody (red); nuclei were labelled with Hoechst dye (blue). B) Total RNA isolated at 1, 6 and 18 hpi was analysed by RT-qPCR to measure levels of IAV NP and NS genome segments. Values were normalized to 18S rRNA levels, and expressed as a ratio of CA/07-MA to parental CA/07. C) Relative levels of total PB2-derived genomic segments (PB2e, light blue) and the full-length PB2 vRNAs (PB2i, blue) were measured by RT-qPCR (18 hpi) using PB2e and PB2i primer pairs, respectively. D) PB2i to PB2e ratios calculated from (C) were plotted. B-D). Error bars represent standard deviation (n=3). * = p value <0.05, Student's t-test.
